# Shared Decision Making in the Treatment of Rectal Cancer

**DOI:** 10.3390/jcm14072255

**Published:** 2025-03-26

**Authors:** Jonathan S. Abelson, Racquel S. Gaetani, Alexander T. Hawkins

**Affiliations:** 1 Department of Colorectal Surgery, Lahey Hospital and Medical Center, Burlington, MA 01805, USA; jonathan.s.abelson@lahey.org (J.S.A.); racquel.s.gaetani@lahey.org (R.S.G.); 2 Section of Colorectal Surgery, Department of Surgery, Vanderbilt University Medical Center, Nashville, TN 37232, USA

**Keywords:** locally advanced rectal cancer, low anterior resection, abdominoperineal resection, watch & wait, nonoperative management, shared decision making, quality of life, bowel function, patient decision aid

## Abstract

**Background/Objectives**: The management of locally advanced rectal cancer has evolved significantly, shaped by advances in multimodal neoadjuvant therapy and a growing emphasis on organ preservation through the watch-and-wait approach. These advancements, however, introduce complex treatment decisions that require careful consideration by both patients and clinicians. **Methods**: This narrative review explores the evolution of the management of locally advanced rectal cancer and the role of shared decision-making in guiding treatment decisions, particularly for patients facing decisions between surgical resection and watch-and-wait. Additionally, it discusses the development of tools to aid in shared-decision making, current challenges in implementing shared decision-making and future directions for improvement patient centered care in locally advanced rectal cancer management. **Results**: Considerations for decision making include anatomical considerations that influence surgical options, the potential benefits and risks of watch-and-wait versus surgical resection of the rectum, and the impact of treatment on bowel, urinary, and sexual function. Additionally, patients must weigh the long-term implications of their choices on quality of life. **Conclusions**: Shared decision-making has emerged as a critical component of patient-centered care and ensures that treatment decisions align with patients’ values and priorities. Given the preference-sensitive nature of the management of locally advanced rectal cancer, shared decision-making plays an important role in helping patients navigate these decisions.

## 1. Introduction

The management of rectal cancer has undergone significant advancements in the past decade. These innovations have reshaped treatment paradigms, offering patients a broader range of therapeutic options for the management of their cancer. When multiple treatment options exist, shared decision-making (SDM) is essential to ensure that clinicians and patients collaboratively choose a treatment plan that aligns with the patient’s values and preferences. SDM is particularly vital in rectal cancer, where treatment decisions carry profound functional, emotional, and quality of life consequences.

One of the primary considerations in rectal cancer management is whether to undergo a low anterior resection (LAR) or abdominoperineal resection (APR), with the choice dependent on tumor location and involvement of the anal sphincter. While LAR preserves sphincter function, it can lead to bowel dysfunction which can impact daily activities and social interactions. Conversely, APR results in permanent colostomy, which may have psychosocial implications. In cases whether patients with locally advanced rectal cancer (LARC) achieve a clinical complete response (cCR) or near complete response (nCR), the watch-and-wait (W&W) approach may be considered, avoiding surgery and its associated morbidities. However, W&W requires intensive surveillance and carries a risk of local regrowth, which necessitates early detection. Furthermore, patients with upper rectal cancer may have the option to omit radiation therapy, and avoiding the sequela of radiation to the pelvis. The complexity of these treatment choices highlights the necessity of SDM, ensuring that patients receive balanced information about risks, benefits, and functional outcomes, allowing them to make choices that align with their personal preferences.

Traditional decision-making models in healthcare have historically been paternalistic, where healthcare providers make treatment decisions on behalf of patients based on their clinical expertise, while patients play a passive role [1]. This approach assumes that clinicians know what is best for the patient and may not fully consider the patient’s preferences, values, or lifestyle. In contrast, modern SDM approaches emphasize a collaborative approach between patients and clinicians. In these models, clinicians present evidence-based treatment options while patients share their preferences, values and concerns allowing for decisions that are informed, mutually agreed upon, and incorporate patient preferences [2]. This approach is particularly valuable in managing diseases, such as LARC, where multiple treatment options exist with comparable efficacy but differing impact on quality of life. Patients who participate in SDM report greater satisfaction, reduced decisional regret, and stronger adherence to treatment plans [3]. Additionally, SDM can improve quality of life by ensuring that treatment aligns with patients’ preferences [4].

Despite the benefits of SDM, barriers to the implementation of SDM in practice remain. Time constraints are a major challenge as SDM requires in-depth discussions which may exceed standard consultation times. Additionally, clinicians’ biases and variability in practice patterns can lead to inconsistent recommendations. Furthermore, varying levels of patient health literacy and language barriers may hinder effective communication, as some patients may struggle to understand complex medical information [5]. Overcoming these challenges requires clinician education, interdisciplinary collaboration, and the development of structured decision aids to facilitate meaningful conversations between patients and clinicians in the management of LARC.

This review examines the evolution of LARC management and the pivotal role of SDM in guiding treatment choices, particularly when multiple treatment options exist. It also explores the challenges hindering the effective implementation of SDM and outlines future directions for enhancing patient engagement in treatment decisions. Furthermore, it emphasizes the importance of patient-centered approaches, such as the development of structured decision aids, to support informed and value-drive decision-making in rectal cancer management.

## 2. Management of Locally Advanced Rectal Cancer

The management of locally advanced rectal cancer (LARC), defined as American Joint Committee on Cancer (AJCC) TNM stage II and III rectal cancer, has undergone significant advancement over time [6,7]. The adoption of a multidisciplinary approach to the management and surveillance of LARC, including neoadjuvant chemotherapy with radiation therapy, high resolution pelvic magnetic resonance imaging (MRI), and the introduction of total mesorectal excision (TME), has significantly advanced how the disease is managed [8,9,10,11,12]. Total neoadjuvant therapy (TNT), which combines neoadjuvant chemotherapy with concurrent chemoradiation therapy prior to surgical resection has improved the oncological outcomes of patients with LARC [9,10,11]. With TNT, 25–50% of patients may achieve a clinical complete response (cCR), defined as no palpable tumor on digital rectal examination, no visible tumor on endoscopy, and no evidence of disease on cross-sectional imaging [13,14,15,16,17]. These observations have led to the emergence of a non-operative management strategy, termed watch-and-wait (W&W), for patients who have achieved a cCR after TNT.

First described in 2004 by Habr-Gama et al., W&W allows for preservation of the rectum as an alternative to surgery, sparing patients from the morbidity associated with surgical resection [18]. W&W involves strict surveillance with digital rectal examinations, endoscopy, and MRI to detect and manage local regrowth [18,19]. By avoiding surgical resection, W&W allows eligible patients to avoid an ostomy and experience improved quality of life (QoL), with better bowel, sexual, and urinary function compared to those undergoing TME [20,21,22].

W&W has gained traction worldwide and is now included in the National Comprehensive Cancer Network (NCCN), American Society of Colon and Rectal Surgeons (ASCRS), and European Society of Medical Oncology (ESMO) rectal cancer guidelines [13,23,24,25,26]. Early studies have demonstrated promising oncologic outcomes, with overall survival rates of 81–92% and disease-free survival of 94–97% [27,28,29] (Table 1). Large multi-institutional trials investigating the outcomes of W&W including the International Watch & Wait Database (IWWD) study found a 2-year local regrowth rate of 25.2% with most regrowth occurring within the first two years, and an 8% rate of distant metastasis [29]. Additionally, studies have demonstrated that in patients who are successfully managed with W&W for 1 year, the probability of remaining free of local regrowth for an additional 2, 3, and 5 years are 88.1%, 97.3%, and 98.6%, respectively [30].

As the oncologic outcomes for patients with LARC have continued to improve, research has focused on identifying the best combination of chemotherapy, radiation therapy and surgery. The RAPIDO trial compared the efficacy of short-course radiotherapy (SCRT) followed by chemotherapy followed by TME to long course radiation therapy (RT) with concomitant chemotherapy followed by TME in patients with high-risk LARC [11]. The SCRT group achieved significantly higher rates of pathologic complete response, lower rates of distant metastasis at 5 years, and lower probability of disease-related treatment failure at 3 years [11,31,32]. More recently, the PROSPECT trial, a multicenter noninferiority randomized control trial, demonstrated non-inferiority in the selective use of RT for the treatment of LARC in patients without high-risk features and who were candidates for sphincter-sparing surgery [33]. The ASCRS Clinical Practice Guidelines for the Management of Rectal Cancer have incorporated the findings from both the RAPIDO and PROSPECT trial to inform treatment recommendations for LARC, reflecting the ongoing evolution in the management of this disease [13].

**Table 1:** Key studies investigating watch-and-wait approach for locally advanced rectal cancer.

Authors/Study (Study Design)	Year	Rectal Cancers Included	N	Treatment Arms/ Neoadjuvant Therapy Regimen	Survival Outcomes %	cCR	LRR	Findings
Habr Gama et al. [18] (Observational retrospective)	2004	cT1-4 N1-2	265 71 W&W	CRT (50.4 Gy/28 fx + 5-FU and leucovorin) → W&W in those with cCR	DFS: 5-year 92% OS: 5-years 100%	27%	5-year 2.8%	There was a locoregional recurrence rate of 2.8% in the W&W group. There was no difference in DFS for those in W&W and those who had an iCR and underwent TME
				CRT → TME in those with iCR	DFS: 5-year 83% OS: 5-year 88%	—	—	
Martens et al. [28] (Prospective Cohort)	2016	Rectal cancer without distant metastasis	100	CRT (1.8 Gy, 28 fx) with capecitabine or 5 Gy for 5 days → assessed for tumor response 8 weeks after completion of RT	DFS: 3-year 80.6% OS: 3-year 96.6%	61% nCR 39%	15%	W&W for cCR and nCR results in high 3-year OS and DFS.
Van der Valk et al. [29] (International multicenter observational mixed prospective and retrospective using IWWD)	2018	Rectal cancer who had cCR are entered into W&W	1009	Various—CRT most common (45 Gy, 50 Gy, 54 Gy or 60 Gy) with capecitabine or 5-FU	DFS: 5-year 94% OS: 5-year 84.7%	—	2-year 25.2%	Those in W&W had high 5-year OS and DFS 31% has local excision and 78% had salvage TME after recurrence
Fernandez et al. [30] (Retrospective multicenter registry study using IWWD)	2021	Rectal cancer who had cCR and managed with W&W alone	793	Various—CRT most common (45 Gy, 50 Gy, 54 Gy or 60 Gy) with capecitabine or 5-FU	Local regrowth-free survival 83.8% Distant metastasis-free survival 97.1%	—	—	Probability of remaining free of local regrowth for 2 years if you have a cCR for 1 year was 88.1%, for 3 years 97.3%, for 5 years 98.6%
OPRA trial [17] (Prospective randomized phase II trial)	2022	Clinical stage II (T3–4, N0)—stage III (any T, N1–2)	324	Induction chemotherapy (FOLFOX or CAPOX) → CRT (4.5 Gy, 25 fx to nodes and 5–5.6 Gy to tumor) with capecitabine or 5-FU → NOM in cCR/nCR	DFS: 3-year 76% OS: 3-year ~95%	71% *	40%	Similar 3-year DFS were observed in those who underwent W&W compared to historical control and 3-year DFS did not differ amongst induction chemotherapy and consolidation chemotherapy. DFS was similar for those undergoing TME for iCR and for TME after re-growth
				CRT (4.5 Gy, 25 fx to nodes and 5–5.6 Gy to tumor) with capecitabine or 5-FU→ consolidation chemotherapy (FOLFOX or CAPOX) → W&W in cCR/nCR	DFS: 3-year 76% OS: 3-year ~95%	76% *	27.5%	

Adapted from [34]. * cCR and nCR; Abbreviations: CAPOX, capecitabine and oxaliplatin; cCR, complete clinical response; CRT, chemoradiotherapy; DFS, disease-free survival; FOLFOX, 5-fluorouracil, leucovorin calcium (folinic acid), and oxaliplatin; fx, fractions; 5-FU, 5-fluorouracil; iCR, incomplete clinical response; IWWD, International Watch & Wait Registry; LRR, locoregional recurrence rates; nCR, near complete clinical response; OS, overall survival; RT, radiation therapy; TME, total mesorectal excision; W&W, watch-and-wait.

## 3. Shared Decision-Making

Shared decision-making (SDM) is a patient-centered approach that fosters collaborative partnership between clinician and patient. Unlike a traditional, paternalistic model of decision-making, where clinicians make decisions based on their expertise and present them to patients, SDM ensures that patients are central to the decision process. Montori and colleagues define SDM as a collaborative process where patients, in partnership with their clinician, are encouraged to consider the available care options and work together to choose the best course of action that aligns with their personal care goals [35]. The SDM model involves several key components [36] (Table 2). First, clinicians define the problem and provide clear, evidence-based guidance regarding treatment options, including the benefits, risks, and potential outcomes of each choice. In parallel, patients are encouraged to express their preferences, values, and lifestyle considerations that may influence the decision. The goal is to arrive at a decision that not only aligns with the best available clinical evidence but also prioritizes the patient’s individual needs and goals of care [37,38].

Elwyn et al. developed a three-talk model for describing the process of shared decision making within a patient clinical encounter [39]. The model breaks the process into three distinct phases—Team Talk, Option Talk, and Decision Talk —that are anchored on the concepts of active listening and deliberation. The initial Team Talk phase focuses on establishing a partnership between the clinician and the patient by creating a supportive environment, introducing the concept of SDM, and ensuring the patient feels empowered to participate. In the second phase, the clinician discusses potential treatment options using risk communication principles with the goal of providing evidence-based guidance to the patient. The provider should facilitate a dialogue that encourages patients to ask questions, clarify any doubts, and ensure they fully understand the implications of each choice. Lastly, in Decision Talk, patient and provider work together to decide on the most appropriate treatment, balancing evidence-based guidance with the patient’s values and goals of care. Ultimately, the three-talk model simplifies the shared decision-making process by providing concrete objectives and ensuring that decisions are well informed and patient-centered.

## 4. Tools to Aid in Shared Decision Making

Shared decision making (SDM) is an important part of surgical treatment [40]. Several methods exist to improve decision making in the surgical sciences. These include communication training for surgeons, educational tools for patients and decision aids to promote shared decision making between the surgeon and the patients. Multiple studies describe both techniques and training programs to improve surgeon communication. These include the best case/worst case framework as well as methods to teach surgical residents [41,42]. Educations tools are readily available in both print form and on-line. Producers of these materials range from governmental agencies to clinical societies and patient groups. Finally, educational decision aids have been previously shown to improve outcomes in surgical diseases such as prostate cancer, breast cancer and joint replacement [43,44,45]. A systematic review by Stacey et al. (2017) in the Cochrane Database of Systematic Reviews highlighted that decision aids for people facing health treatment or screening decisions are associated with higher patient knowledge, more accurate risk perceptions, greater alignment between patient values and choices, and reduced decisional conflict. Although the primary focus was on decision quality and patient satisfaction, some studies within the review suggested potential links to improved health outcomes and quality of life (QoL) [46]. A study by van Tol-Geerdink et al. explored the use of decision aids in prostate cancer surgery [47]. The findings indicated that patients who used decision aids reported better QoL metrics post-surgery, attributing this to more informed and value-congruent decisions that likely reduced post-treatment regret and psychological distress. While direct links to survival were not established, improved QoL is a critical component of overall patient well-being. research on decision aids in breast cancer surgery by Waljee et al. (2007) found that patients who used these tools experienced better QoL outcomes, including lower anxiety and depression levels [48]. The study underscored the importance of aligning surgical choices with patient preferences, which can significantly impact postoperative QoL. While direct evidence linking decision aids to improved survival is limited, the indirect benefits of enhanced decision quality and patient satisfaction can contribute to better adherence to treatment plans, timely interventions, and improved overall health management. Consequently, these factors can positively influence long-term health outcomes and survival rates. Decision aids in colorectal surgery have also been developed, most recently in diverticulitis [49].

A well-established decision aid for surgical diseases that has shown a significant impact on patient-reported outcomes is the “Ottawa Personal Decision Guide” (OPDG). This decision aid is designed to support patients in making informed, value-based decisions regarding their surgical options. The OPDG helps patients clarify their values, assess the benefits and risks of different surgical interventions, and consider their personal preferences. Studies have demonstrated that using the OPDG enhances patient knowledge, reduces decisional conflict, and leads to decisions that align more closely with patients’ values and preferences [50]. Additionally, the OPDG has been associated with improved patient satisfaction and better alignment of surgical decisions with desired health outcomes, thereby positively impacting patient-reported outcomes [46]. These findings underscore the importance of utilizing validated decision aids in clinical practice to facilitate shared decision-making and improve the overall quality of care for patients facing surgical decisions.

## 5. Shared Decision Making in Rectal Cancer

Shared decision making (SDM) requires the integration of evidence based medicine with patients’ values and preferences to facilitate discussion between patients and clinicians, ultimately guiding them towards the treatment option that best aligns with their social, economic, and personal preferences [51]. For patients with rectal cancer, there are several preference-sensitive decision points to be considered during the decision-making process based on history, physical examination, response to treatment, tumor histology, oncological outcomes, and patient quality-of-life (QoL) preferences.

### 5.1. Low Anterior Resection vs. Abdominoperineal Resection

Anatomical considerations play a role in determining whether patients require a low anterior resection (LAR) or abdominoperineal resection (APR) at the time of surgery. NCCN guidelines recommend APR when the tumor involves the anal sphincter, levator muscles, or when a negative margin resection would result in loss of anal sphincter function and therefore fecal incontinence [23]. Each procedure is associated with morbidity that can impact QoL (Table 3). Patients undergoing LAR often report bowel dysfunction including fecal incontinence, frequent bowel movements, urgency, and/or incomplete emptying, collectively referred to as low anterior resection syndrome (LARS) [52,53,54,55]. The presence of LARS symptoms is negatively associated with QoL, and may impact patients’ daily activities and social interactions [56]. LARS is categorized into three severity levels, no LARS, minor LARS, and major LARS, based on the LARS score and symptom severity [57]. A cross-sectional study by Pieniowski et al. found that patients with LARS were more likely to be younger, had low tumor levels, and undergo preoperative radiotherapy compared to those with no LARS. Furthermore, among patients with LARS, those with major LARS reported significantly worse global health status, reduced role and social functioning, and more severe symptoms including nausea, pain, and diarrhea [58].

Patients who undergo APR tend to report fewer gastrointestinal symptoms compared to those undergoing LAR, but require permanent colostomy which may be associated with morbidity [59,60,61,62]. Fortunately, overall QoL tends to be similar between those undergo LAR and APR, however, specific domains of QoL differ. Patients who undergo APR report better cognitive and social function while those undergoing LAR have improved physical function [53,54,63]. A multivariate analysis of QoL in those undergoing APR found that patient factors such as male sex, young, age and the perception that surgery led to sexual inactivity to be independently associated with worse long-term QoL [64].

Additional QoL considerations that can influence surgical decisions include whether patients with baseline fecal incontinence could benefit more from an APR to prevent the exacerbation of incontinence that may be exacerbated with LAR, irrespective of tumor location. The literature remains mixed regarding the comparative sexual outcomes of LAR and APR. While some studies suggest better sexual function following LAR, others indicate no significant difference, particularly in patients who underwent neoadjuvant RT [32,65,66].

### 5.2. Proctectomy vs. Watch & Wait in Patients with Clinical Complete Response

The decision to pursue watch-and-wait (W&W) or surgical resection of the rectum is highly individualized. It requires a multidisciplinary approach involving the patient, their healthcare team, and support network. SDM plays a pivotal role in ensuring that the patient’s values, preferences, and priorities are considered during treatment.

Several critical factors influence the decision between W&W and surgery including tumor location, the effects of pelvic radiation therapy (RT), and the potential impacts of surgery on QoL. Total mesorectal excision (TME), whether performed as an APR or LAR, may be associated with adverse surgical outcomes. These include bowel, sexual, and urinary dysfunction, as well as the need for temporary or permanent ileostomy or colostomy. As mentioned previously, each surgical approach has distinct considerations that my impact QoL differently. Additionally, surgery carries the risk of short- and long-term complications such as anastomotic leak, wound infection, bleeding, ileus, stoma-related issues, and gastrointestinal dysfunction such as incontinence or LARS.

For patients who achieve a clinical complete response (cCR), opting for W&W offers the opportunity to avoid proctectomy and consequently avoid the associated surgical morbidity. Still, these patients will almost always receive RT which in and of itself may contribute to gastrointestinal symptoms, including incontinence, difficulty with evacuation, and frequency which may have impacts of QoL [67,68,69,70,71]. RT can also have effects on urinary and sexual function further compromising QoL [71,72,73,74]. A study by Pollack at el., utilizing patients from the Stockholm rectal cancer trials, found that up to 45% of patients may experience urinary incontinence associated with RT, with men disproportionately affected at a higher rate [73]. Sexual dysfunction is similarly more common in men and may include erectile dysfunction and retrograde ejaculation, while women may experience vaginal dryness and dyspareunia [65,75,76]. Studies indicate that up to one-third of patients who enroll in W&W may experience major LARS symptoms after total neoadjuvant therapy (TNT) [66]. Despite this, evidence suggests that patients who are managed with W&W generally report superior QoL and functional outcomes compared to those who undergo TME [21,77,78]. A matched-controlled study by Hupkens at al. further supports this, showing that non operative management is associated with superior QoL and have fewer rates of defecation problems, sexual dysfunction and urinary dysfunction [20].

Further considerations include the need to balance oncologic safety with patients’ values, as some individuals may prioritize QoL over the risk of cancer recurrence or progression. Patients must also understand the surveillance requirements associated with W&W, as this approach necessitates close monitoring for recurrence as the ability to detect regrowth early is critical to the oncologic success of this approach [29,79]. These considerations underscore the complexity of rectal cancer management and the importance of SDM to optimize both oncologic and functional outcomes for patients. It is important to counsel patients on the risks, benefits, and considerations of both options so that patients can make an informed decision.

Personalized assessments can further enhance SDM by considering patient specific factors. Emerging data suggests predictors of a cCR to TNT, helping identify patients who may be more likely to achieve a cCR and therefore benefit from W&W approach. Patients who achieve a cCR tend to be younger, have normal carcinoembryonic antigen (CEA) levels, have clinically node-negative disease, and smaller tumors [80]. Additional predictors, such as low rectal tumors and absence of extramural vascular invasion are also associated with cCR and pathologic complete response [81]. This finding suggests that individualized prediction models could provide patients with more tailored insights into their specific likelihood of success with W&W.

Beyond clinical assessment, understanding patient knowledge, health literacy, and decision-making preferences is equally as important in improving SDM. Patients vary widely in their ability to process medical information and health literacy influences the comprehension of complex concepts such as recurrence risks, surveillance protocols and treatment trade-offs [82,83]. Assessing a patient’s knowledge allows for more personalized treatment discussions to improve comprehension. Improved patient knowledge has been shown to improve satisfaction and reduce decisional regret [84]. Furthermore, preference assessment tools, such as the Control Preference Scale, can help determine the level of involvement a patient desires in their treatment decisions [85]. Some patients prefer full decision-making autonomy while others prefer a more physician-guided approach. Recognizing these differences allows for more effective and individualized SDM discussions.

Another critical aspect is risk aversion, as patients differ in their willingness to accept oncologic uncertainty in exchange for improving quality of life [86]. Integrating risk preference assessment into SDM discussions can help clinicians to better understand how patients interpret and weigh risks, ensuring that treatment recommendations align with their priorities. Incorporating SDM principles in these cases can also help mitigate anxiety by ensuring that patients fully understand their prognosis, treatment options, and the likelihood of recurrence or complications [4,87].

### 5.3. Neoadjuvant Regimen for Patients with Upper Rectal Cancer

Tumor location, while important in surgical decision making also has important implications in determining prognosis and neoadjuvant treatment regimens. Rectal cancers are defined anatomically as upper, middle, or lower rectal tumors based on their distance from the anal verge. According to the National Comprehensive Cancer Network (NCCN), the rectum is defined as extending up to 12 cm from the anal verge, with upper rectal tumors located between 10–12 cm, middle rectal tumors between 5–10 cm, and lower rectal tumors within 5 cm of the anal verge [23].

Historically, RT was found to be most efficacious for middle to lower rectal cancers with less clear benefit for upper rectal cancers [88]. Both the American Society of Colon and Rectal Surgeons (ASCRS) and NCCN guidelines indicate that for patients with T3N0 rectal cancer of the upper rectum, initial surgical resection without neoadjuvant chemoradiotherapy is appropriate, as the risk of local recurrence in the upper rectum is less than those of the mid to lower rectum [13,23]. This approach then reduces the potential treatment-related toxicities of RT without compromising oncologic outcomes.

The effects of RT should be balanced with the risk of urinary and sexual function in patients who undergo surgery as TME may lead to inadvertent autonomic nerve damage exacerbating these issues regardless of whether LAR or APR is performed. A systematic review found that sexual dysfunction may occur in 23–69% of men and 12–62% of woman after proctectomy, with those undergo neoadjuvant RT having poorer sexual function compared to those without RT [71,72,89,90].

The question of whether RT provides a benefit in patients with upper rectal cancer, combined with concerns about its potential toxicity, motivated the design and execution of the PROSPECT trial [33,91]. This multicenter, randomized, non-inferiority trial evaluated the selective use of RT in patients with locally advanced rectal cancer (LARC) who were eligible for sphincter-sparing surgery. The findings of this trial have further refined the treatment strategies for LARC in selected patients showing that the omission of RT does not compromise 5-year disease free survival. Additionally, patients who underwent chemotherapy alone reported significantly better overall bowel function and fewer rates of fatigue and sexual dysfunction which effectively was able to minimize the morbidity associated with pelvic RT without compromising oncologic outcomes [33,91].

**Figure 1:**
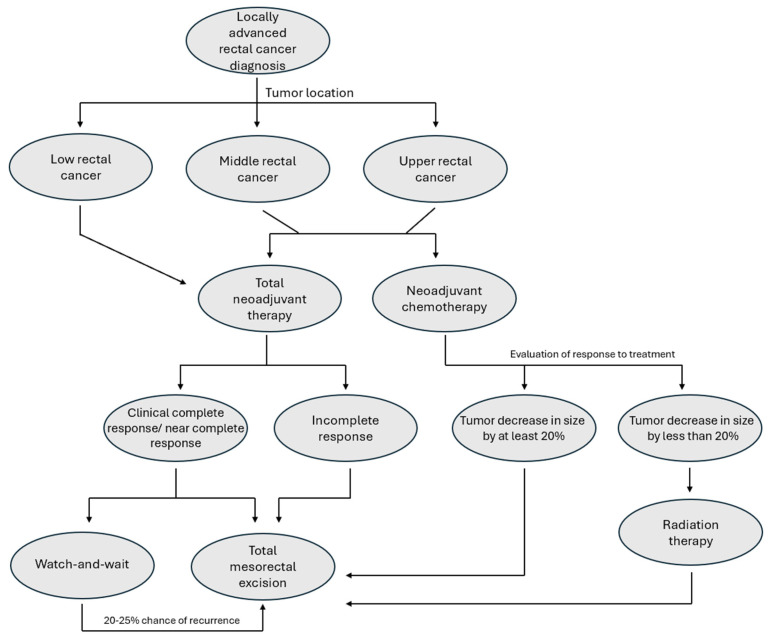
Treatment decisions in the management of locally advanced rectal cancer.

The findings of this trial as well as ongoing trials further add to the complexity of both surgeon and patient decision-making for rectal cancer (Figure 1). Furthermore, it is unclear how this trial will impact decision making in clinical settings at the time of this writing.

### 5.4. Local Excision with Radiation Therapy vs. Proctectomy in Patients with Stage 1 Rectal Cancer

There is an unusual paradigm whereby patients with stage II and III rectal cancer may be afforded the option for organ preservation whereas patients with stage I rectal cancer are traditionally offered proctectomy with the goal of potentially avoiding chemotherapy and RT. This prompted investigation into whether local excision plus RT may serve to be an effective treatment algorithm to allow for organ preservation in early-stage rectal cancers as compared to upfront proctectomy.

There have been several studies that have investigated this in several iterations [92,93,94,95,96]. The ACOSOG Z6041 trial, a multi-institutional single-arm feasibility study, demonstrated an improved 3-year disease free survival in the intention-to-treat group. This trial concluded that organ-preservation is feasible in patients with T2N0 distal rectal cancer, though careful patient selection is emphasized [95]. More recently, the TREC trial randomized patients with early-stage rectal cancer to either SCRT followed by transanal endoscopic microsurgery (TEMS) or TME. The study found that organ preservation was achievable in 56% of patients at 30 months, with similar overall and disease-free survival rates between groups. However, the TEMS group exhibited a higher 3-year local recurrence rate compared to those who underwent proctectomy.

**Figure 2:**
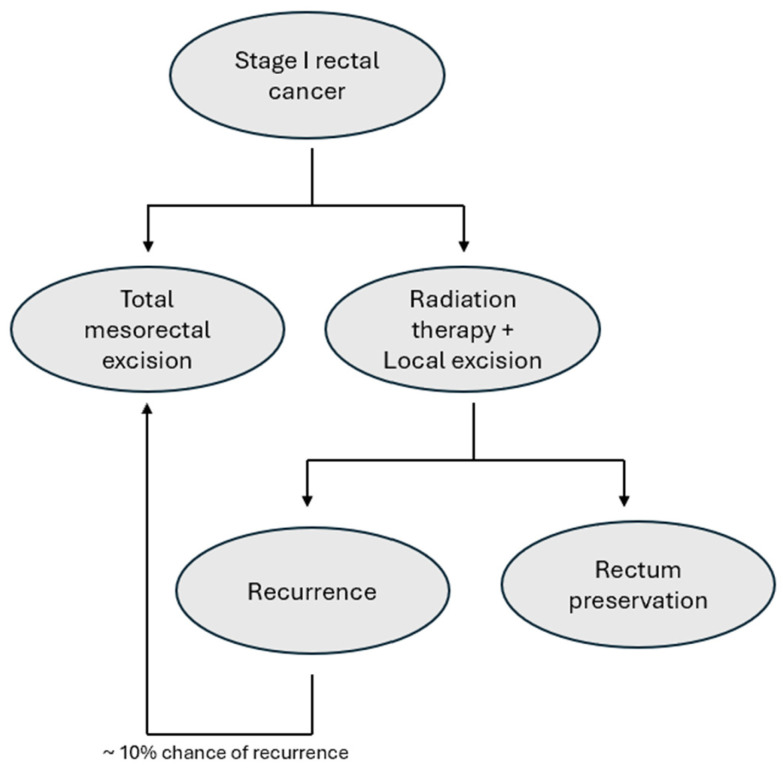
Treatment decisions in the management of early-stage rectal cancer.

Local excision combined with RT presents a viable alternative to proctectomy for stage I rectal cancer (Figure 2). However, these options come with important trade-offs, including the potential toxicities of RT and chemotherapy as well as an increased risk of local recurrence associated with local excision. These considerations emphasize the importance of SDM in developing a personalized management plan for rectal cancer. A qualitative study by Rubens et al. highlighted the importance of SDM in the context of stage I rectal cancer, noting that both clinicians and patients recognized the importance and value of SDM [97]. Patients emphasized the need for more comprehensive information to make informed decisions about their care. This underscores the importance of clear clinician communication that provides balances information about treatment options so that patients can then consider their priorities and make a decision that aligns with their goals.

## 6. Future Directions in Shared Decision Making in Rectal Cancer

Overall, patients with rectal cancer are faced with medically complex decisions that require several considerations with impacts on quality of life (QoL). Several decision aids have been validated for rectal cancer patients. Wu et al., developed a decision aid to inform patients about the differences between low anterior resection (LAR) and abdominoperineal resection (APR), including their respective benefits and risks [98]. Results of this study demonstrate that the patient decision aid (PtDA) was able to increase patient knowledge by 37.5% from baseline and reduce decisional conflict by 24.2%. Additionally, 96% of participants would recommend the use of this tool to others [98]. Additionally, a Belgium study by Smets et al., successfully developed a decision aid for rectal cancer patients who have achieved a clinical complete response (cCR) after total neoadjuvant therapy (TNT) to guide decision making. In this investigation, patients with rectal cancer and surgery provider collaborated to develop a PtDa using the Ottawa Decision Support Framework and international patient decision aid standards (IPDAS). The majority of patients found this tool valuable in the decision making process [99]. No such tool is available in the United States.

Recent advancements in digital decision aids, such as artificial intelligence (AI) -based tools and mobile applications, have shown promise in improving shared decision-making for rectal cancer patients, particularly in terms of personalized risk assessment, treatment selection, and patient engagement. A study by Yang et al. explored the use of AI-based decision support systems in the management of rectal cancer [100]. The system integrated clinical data, imaging, and pathological findings to provide personalized risk assessments and treatment recommendations, which facilitated more informed and tailored decision-making processes for patients and clinicians. Additionally, mobile applications designed to support rectal cancer patients have demonstrated significant improvements in patient engagement and satisfaction. A study by Dao et al. evaluated a mobile app that provided patients with real-time information about their condition, treatment options, and potential outcomes [101]. The app also included interactive features to help patients clarify their values and preferences, which were then used to guide shared decision-making discussions with their healthcare providers. The results indicated that patients using the app reported higher levels of understanding, reduced decisional conflict, and increased confidence in their treatment choices. Overall, the integration of digital decision aids in the management of rectal cancer has shown potential to enhance personalized risk assessment, improve treatment selection, and foster greater patient engagement, ultimately leading to more effective and patient-centered care.

To aid in the decision-making process of patients with locally advanced rectal cancer (LARC) who have achieved a cCR and are eligible for watch-and-wait (W&W), a PtDA is currently undergoing validation via qualitative interviews with patients and surgeons through a multicenter collaboration within the United States. Development of the instrument is in alignment with the Ottawa Decision Support Framework and the IPDAS. The PtDA integrates evidence-based information on oncological outcomes, risks, benefits, and QoL considerations associated with W&W compared to surgical intervention. Additionally, the tool includes a dedicated section for patients to reflect on their personal values and preferences, alongside an interactive feature to explore how various factors may influence their treatment decisions.

The primary objective of the PtDA is to present clinical data in an accessible and comprehensible format, enabling patients and their families to better understand the multifaceted considerations involved in the decision-making process. Effectively educating patients and managing their expectations are crucial to ensuring they are fully informed about the potential effects of RT, surgery, and non-operative management.

While a PtDA holds significant promise for supporting decision-making in this patient population, current practice patterns for W&W and neoadjuvant therapy regiments are poorly understood. Societal and national guidelines do not define the optimal candidate for W&W, and therefore, patient selection is provider and institution dependent.

The management and treatment of LARC is becoming increasingly nuanced as oncological and QoL considerations evolve alongside advancements in treatment modalities. The development and validation of a PtDA represents a clinical step toward empowering patients with evidence-based tools that facilitate SDM. By aligning treatment decisions with patient priorities, providers can optimize both clinical outcomes and patient satisfaction.

However, the dynamic and ever-changing landscape of rectal cancer necessitates a PtDA that is adaptable to emerging research and evolving treatment paradigms. This tool must be designed to incorporate input from surgeons reflecting diverse practice patterns in order to ensure broad applicability. Moreover, as treatment strategies continue to evolve, particularly with the growing interest in de-escalation therapies and alternative management approaches, the PtDA must remain flexible, incorporating the latest evidence in order to stay relevant and valuable to patients and clinicians.

To ensure the effective application of W&W and other treatment strategies for rectal cancer, further efforts are needed to standardize guidelines, better understand practice variations, and continually refine patient materials. By fostering national collaboration among clinicians and introducing a PtDA, the complexities of LARC management can be more effectively navigated.

## 7. Conclusions

As treatment options for rectal cancer continue to evolve, so does the complexity of decision-making for both patients and surgeons. Patients with rectal cancer face multiple preference-sensitive decisions, including choices between low anterior resection and abdominoperineal resection, surgical resection versus watch-and-wait and the selective use of radiation therapy based on tumor location and risk profile. Each of these decisions carries significant implications for quality of life, particularly in relation to bowel, urinary and sexual function. In this context, shared decision-making is essential and valuable as it enhances patient satisfaction and outcomes. Therefore, optimizing care requires more than clinical expertise; it necessitates a robust understanding of individual patient preferences, values, and lifestyle priorities. Tools such as patient decision aids and communication training for surgeons offer promising avenues to support more effective and individualized decision-making by fostering shared decision-making.

## Figures and Tables

**Table 2:** Essential elements of shared decision making.

Essential Element	Notes
Define/explain problem	In order for shared-decision making to occur, patients and providers must first define and/or explain the problem that needs to be addressed.
Present options	Physicians should review options if options exist, and patients should raise options of which they may be aware.
Discuss Pros/cons	Particularly important as physicians and patients may hold different perspectives on the relative importance of benefits, risks, and costs, including convenience and opportunity cost.
Patient values/preferences	Includes ideas, concerns, and outcome expectations—as well as physician knowledge and recommendations in the context of the decision at hand.
Check understanding	Throughout the process, both parties should periodically check understanding of facts and perspectives, providing further clarification as needed.
Make or explicitly defer decision	Decisions are not always ‘‘made’’ when problems are first discussed; they may be explicitly deferred for a later time (e.g., pending discussion with members of the family and/or healthcare team).

**Table 3:** Key studies investigating quality of life and functional outcomes between abdominoperineal resection and sphincter-preserving surgery/low anterior resection for rectal cancer.

Authors/Study (Study Design)	Year	Rectal Cancers Included	N	F/u Time	QoL Assessment	Functional Outcome Assessment	Findings
How et al. [52] (Prospective cohort)	2012	Adenocarcinoma of the low rectum (within 6 cm of the anal verge)	62	2 yrs	EORTC QLQ-C30, EORTC QLQ-CR28, Coloplast stoma QoL	ST. Mark’s bowel function	No significant difference in global QoL at 1 and 2 years. APR: better cognitive and social functioning, less pain, sleep disturbance at 1 year LAR: better sexual function at 1 year 72% experience a degree of fecal incontinence though this mostly improved at 2 years.
Russell et al. [55] (RCT)	2015	Adenocarcinoma of rectum located within 12 cm from anal verge	987	1 yr	FACT-C and EORTC-QLQ-C38	─	No difference in FACT-C scores 1 year after surgery. No difference in male or female sexual dysfunction. SSS: Better body image APR: Worse GI symptoms
Koëter et al. [53] (Longitudinal prospective population-based survey)	2019	Colorectal cancer survivors 1–11 years after diagnosis	905	─	EORTC QLQ C30 and EORTC QLQ-CR38	─	LAR: better physical functioning, body image, male sexual function. Did not change with time No difference in physical functioning in those with stoma who underwent APR or LAR though those who underwent APR reported better body image and fewer stoma related problems than those with stoma who underwent LAR.

Abbreviations: APR, abdominoperineal resection; EORTC, European Organization of Research and Treatment of Cancer; FACT-C, Functional Assessment of Cancer Therapy for patients with colorectal cancer; F/u, follow-up; GI, gastrointestinal; LAR, low anterior resection; QoL, quality of life; RCT, randomized control trial; SSS, sphincter sparing surgery; yr(s), year(s).

## Data Availability

The data presented in this study are available on request from the corresponding author.

## List of Abbreviations

AI	Artificial intelligence
AJCC	American Joint Committee on Cancer
APR	Abdominoperineal resection
ASCRS	American Society of Colon and Rectal SUrgery
CEA	Carcinoembryonic antigen
cCR	Clinical complete response
ESMO	European Society of Medical Oncology
IPDAS	International patient decision aid standards
LAR	Low anterior resection
LARC	Locally advanced rectal cancer
LARS	Low anterior resection syndrome
MRI	Magnetic resonance imaging
NCCN	National Comprehensive Cancer Network
nCR	Near complete response
OPDG	Ottawa Personal Decision Guide
ptDA	Patient decision aid
RT	Radiation therapy
SCRT	Short-course radiation therapy
SDM	Shared decision-making
TEMS	Transanal endoscopic microsurgery
TME	Total mesorectal excision
TNT	Total neoadjuvant therapy
QoL	Quality of life
W&W	Watch-and-wait
